# Conceptualizing ecosystem tipping points within a physiological framework

**DOI:** 10.1002/ece3.3164

**Published:** 2017-06-28

**Authors:** Christopher D. G. Harley, Sean D. Connell, Zoë A. Doubleday, Brendan Kelaher, Bayden D. Russell, Gianluca Sarà, Brian Helmuth

**Affiliations:** ^1^ Department of Zoology and Institute for the Oceans and Fisheries University of British Columbia Vancouver British Columbia Canada; ^2^ Southern Seas Ecology Laboratories School of Biological Sciences & Environment Institute University of Adelaide Adelaide South Australia Australia; ^3^ National Marine Science Centre & Centre for Coastal Biogeochemistry Research School of Environment, Science and Engineering Southern Cross University Coffs Harbour New South Wales Australia; ^4^ The Swire Institute of Marine Science School of Biological Sciences The University of Hong Kong Hong Kong Hong Kong; ^5^ Laboratorio di Ecologia Sperimentale Dipartimento di Scienze della Terra e del Mare Università degli Studi di Palermo Palermo Italy; ^6^ Department of Marine and Environmental Sciences and School of Public Policy and Urban Affairs Northeastern University Boston MA USA

**Keywords:** food web dynamics, multiple stressors, performance curves, phase shifts, physiological stress, species interactions

## Abstract

Connecting the nonlinear and often counterintuitive physiological effects of multiple environmental drivers to the emergent impacts on ecosystems is a fundamental challenge. Unfortunately, the disconnect between the way “stressors” (e.g., warming) is considered in organismal (physiological) and ecological (community) contexts continues to hamper progress. Environmental drivers typically elicit biphasic physiological responses, where performance declines at levels above and below some optimum. It is also well understood that species exhibit highly variable response surfaces to these changes so that the optimum level of any environmental driver can vary among interacting species. Thus, species interactions are unlikely to go unaltered under environmental change. However, while these nonlinear, species‐specific physiological relationships between environment and performance appear to be general, rarely are they incorporated into predictions of ecological tipping points. Instead, most ecosystem‐level studies focus on varying levels of “stress” and frequently assume that any deviation from “normal” environmental conditions has similar effects, albeit with different magnitudes, on all of the species within a community. We consider a framework that realigns the positive and negative physiological effects of changes in climatic and nonclimatic drivers with indirect ecological responses. Using a series of simple models based on direct physiological responses to temperature and ocean *p*CO_2_, we explore how variation in environment‐performance relationships among primary producers and consumers translates into community‐level effects via trophic interactions. These models show that even in the absence of direct mortality, mismatched responses resulting from often subtle changes in the physical environment can lead to substantial ecosystem‐level change.

## 1 INTRODUCTION

Global climate change is often considered as a multi‐layered stressor, eliciting a range of highly nonlinear responses in biological systems (Doney et al., 2012). A major emphasis of forecasting approaches is thus to understand how multiple stressors interact to drive patterns of ecosystem‐level stability (Isbell et al., 2015), or instability (Drake & Griffen, 2010; Lubchenco & Petes, 2010), conceptualized as phase shifts and tipping points. Yet, an increasing number of studies are showing just how difficult forecasting community and ecosystem‐level responses to changes in multiple climatic‐ and nonclimatic factors can be (Pawar, Dell, & Savage, 2015). Of continual surprise has been the unexpected ways multiple environmental drivers combine; that is additively, synergistically, or antagonistically (Crain, Kroeker, & Halpern, 2008), and the lack of predictability surrounding those outcomes.

Ultimately, community and ecosystem‐level responses are assumed to be an emergent result of the direct effects of environmental change on the physiology, behavior and survival of individual organisms (Gunderson, Armstrong, & Stillman, 2016; Gunderson & Leal, 2016), which in turn determine indirect interactions that propagate or buffer change to population dynamics and community structure (Ghedini & Connell, 2017; Post, 2013; Seebacher & Franklin, 2012). Yet, seldom are these two divergent scales of approach rectified. Instead, conceptualizations of “environmental stress” at ecosystem scales tend to ignore the ways in which environmental change affects sublethal organismal responses (but see Gutschick & BassiriRad, 2003; Smith, 2011). As we explore in more detail below, most factors typically categorized as “stressors” are at a physiological level biphasic, with abiotic changes exerting negative effects at some levels, and positive physiological effects at others. Importantly, the sensitivity to changes can vary among interacting species so that, for example, an increase in temperature can have a positive impact on one species, while simultaneously negatively impacting individuals of another species within the same assemblage (Kordas et al. 2011; Monaco & Helmuth, 2011).

In contrast, at ecological scales, “environmental stress” is typically considered as a relative quantity (e.g., either “harsh” or “benign”) that affects entire ecosystems (Cheng & Grosholz, 2016; Hart & Marshall, 2013). Thus, for example, suites of interacting species are often considered to respond to anomalous conditions in lockstep (e.g., Stuart‐Smith et al., 2015). This outlook may in some cases stem from the implicit (but recognized as flawed, Stillman & Somero, 1996) assumption that all organisms are perfectly adapted to the environmental conditions they currently experience, and thus any change must be for the worse; the magnitude of the disturbance is thus quantified as the extent to which conditions deviate from the norm (Smith, 2011). This assumption underpins much work on “stress gradients” across space and time, which remains a common feature of many biogeographic studies (McAfee, Cole, & Bishop, 2016) and is formalized as the stress‐gradient hypothesis (Bertness & Callaway, 1994; He, Bertness, & Altieri, 2013; Lortie & Callaway, 2006). The “harsh vs. benign” usage of stress is thus often derived independently of the organisms being affected, or assumes no species turnover across the gradient, which may cloud our understanding of “stress” in the real world (Wood, Lilley, Schiel, & Shurin, 2010). Comparably, ecological phase shifts are generally assumed to occur when environmental control variables exceed some threshold (Connell et al., 2017), but the actual mechanisms driving these assemblage‐level responses are often unknown (Liu, Kattel, Arp, & Yang, 2015).

Previous authors have pointed to the underlying physiological basis of tipping points, and have pointed to differential vulnerability of interacting species, primarily in relation to differences in mortality rates (Gutschick & BassiriRad, 2003; Smith, 2011). Under such scenarios, the magnitude of an environmental change is scaled to the tolerance threshold of each species, and one by one species march off of their respective physiological cliffs; whether or not the extinction of a population has an overall impact on ecosystem function depends on that species' ecological role, for example, as a keystone or foundational species (Allen & Breshears, 1998). While Environmental Stress Models (Bruno, Stachowicz, & Bertness, 2003; Menge & Sutherland, 1976) also recognize that responses to stress can vary among interacting species (e.g., consumers and prey) they too generally consider only differences in the magnitude of stress acting on the different species.

These conceptualizations of environmental stress are therefore at odds with our understanding of how environmental change plays out at the level of organismal physiology, and particularly with sublethal impacts of environmental change on processes such as metabolic demand and productivity. Perhaps not surprisingly, the incorporation of these many complexities into a comprehensive theoretical framework has to date remained elusive. By developing a more realistic framework, we seek to integrate findings across multiple studies and link processes at organismal scales through much larger ecological and biogeographic spatial scales. To develop a more comprehensive view of change, we need to incorporate: (1) the nonlinear, biphasic nature of climatic driver–physiological response relationships, which can be both positive and negative; (2) not only lethality but also sublethal physiological responses; and (3) the ways in which differential physiological responses among interacting organisms indirectly mediate outcomes via interspecific interactions, often in ways that oppose the direct environmental effects (Post, 2013). We, therefore, consider a conceptual realignment of the physiological basis of responses to “climatic stressors” and how intact communities will respond to changes in the physical environment. We present a framework for investigation that is sensitive to variation in physiological responses of producers and consumers to environmental change and their mediation of the supply and use of food resources, which in turn determines community state and vulnerability to perturbation.

## 2 STRESSORS, RESOURCES, AND THE COST‐BENEFIT CONTINUUM

The term “stress” is often defined loosely, with several authors (e.g., Boonstra, 2013; McEwen & Wingfield, 2010; Schulte, 2014) pointing to inconsistencies in its use among scales of exploration as diverse as biochemical reactions, whole organisms, and ecosystems. At organismal levels, physiological indicators of stress are classically thought of as measures of an organism's ability to maintain homeostasis in the face of otherwise destabilizing environmental change (Gunderson et al., 2016; Wingfield & Kitaysky, 2002), although authors have also pointed to difficulties with this definition given the highly dynamic nature of most organisms' life histories (McEwen & Wingfield, 2010).

**Figure 1:**
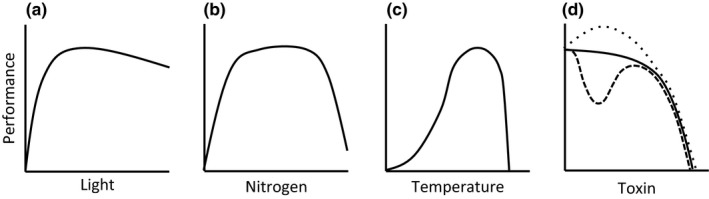
Curves describing physiological performance as a function of (a) light, (b) nitrogen, (c) temperature, and (d) a generic toxin (Vandenberg et al., 2012). Most environmental drivers have complex relationships with organismal performance that include changes in the slope and direction of the effect over certain ranges. Light (a) and nitrogen (b) are both resources necessary for plant growth, but they can both inhibit function if provided in sufficient quantity. The relationship between temperature (c) and performance is famously unimodal. Toxins, often considered as the ultimate “stressor,” may have solely negative effects as concentrations increase (solid line). However, in some cases complex relationships exist between toxin concentration and performance (dotted and dashed lines), as is seen with exposure of *Daphnia* to trinitrotoluene (TNT; Stanley et al. 2013). In this case, a hormetic response is observed (dotted line), and exposures to small levels of TNT lead to an increase in size and reproductive output

In contrast, resources like light and nutrients that are frequently in limiting supply are generically categorized as “resources,” and more is often assumed to be better up to some reasonable threshold. But physiologists have long recognized that this simplified dichotomy between “stressor” and “resource” is inaccurate, and the true impacts of environmental drivers on physiological performance fall on nonlinear continua where both positive and negative effects are possible (Figure 1). For example, moving from darkness into light can clearly benefit a plant, but light can increase to the point where photoinhibition occurs, sometimes at even very low levels for shade‐adapted organisms. Thus, an increase in light intensity can have positive or negative effects depending on intensity level and the photosynthetic physiology of the organism in question (Figure 1a). Similarly, nutrients such as nitrogen are required for growth, but in high concentrations can lead to nutrient toxicity, such that performance of primary producers generally peaks at intermediate nutrient concentrations (Pilon‐Smits, Quinn, Tapken, Malagoli, & Schiavon, 2009; Figure 1b). Even some toxins can have beneficial effects at very low doses and others exhibit complex nonlinear effects based on concentration and an organism's ability to counteract the negative impacts (Calabrese & Baldwin, 2003; Vandenberg et al., 2012; Figure 1d).

On the whole, most environmental drivers–whether generically classified as “stressors” or “resources”–exhibit positive effects at some levels and negative effects at others, with potentially complex relationships between the driver and physiological performance. This type of relationship is particularly well explored for temperature (Figure 1c), and described using a thermal performance curve (Dell, Pawar, & Savage, 2013; Kingsolver & Woods, 2016; Sinclair et al., 2016). Thermal performance curves describe the relationship between temperature and some response assumed to be related to the organism's performance, such as aerobic scope, feeding rate, sprint or swimming speed, growth rate or reproduction (reviewed in Sinclair et al., 2016). Usually, these curves are unimodal and often left‐skewed (Angilletta, 2009) showing a gradual increase in performance with increasing body temperature up to some optimum, above which performance declines rapidly with further temperature increases (Figure 1c).

Allowing for both positive and negative impacts due to changes in environmental conditions differs notably from other approaches that consider only degrees of physiological stress as the result of exposure to environmental change (Doney et al., 2012; Geyer et al., 2011), or ones that assume that physiological responses such as metabolic rate only increase with increasing temperature (e.g., Metabolic Theory of Ecology; Brown, Gillooly, Allen, Savage, & West, 2004). In particular, an expanded definition would allow for an understanding of the ranges over which abiotic variables which can be limiting due to supply (e.g., carbon, nitrogen or light at low levels), or are limiting *via* physiological stress effects (e.g., nitrogen or light at very high levels or temperatures at low or high extremes). Critically, the aspects of environmental change that are considered “stressful” depend on the shape of, and relative position on, each species' physiological performance curve and cannot, therefore, be considered without reference to the organisms being affected (Torossian, Kordas, & Helmuth, 2016). *As a consequence, “stress” cannot be defined simply on the basis of environmental conditions alone*. And, because the relationship between environment and performance varies among species (and even among individuals), a change in level that would be considered as stressful for one species may well benefit another. This context provides a means of considering the impacts of environmental change on consumers and their resources (among many other potential species interactions), an idea that we explore in detail below.

## 3 CONSIDERING PERFORMANCE RESPONSES IN THE CONTEXT OF INTERACTING SPECIES

**Figure 2:**
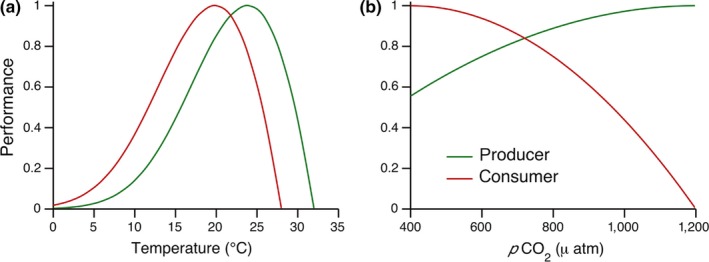
Species vary in their physiological responses to abiotic factors such as (a) temperature and (b) *p*CO_2_. Green lines indicate a hypothetical primary producer, and red lines represent a hypothetical consumer. When such relationships differ among interacting species, relative performance levels change with absolute value of the environmental driver

Organisms–even those living in the same assemblage–can display marked differences in performance curves, including the breadth of the curve, the degree of skewness and the position of the optimum (Angilletta, Niewiarowski, & Navas, 2002; Dell et al., 2013; Pawar, Dell, Savage, & Knies, 2016). A simple example of this, involving a difference in the position of the thermal optimum between two species, is diagrammed in Figure 2a. Environmental change may also affect species differently because of fundamental differences in the driver's mode of action. The absorption of atmospheric CO_2_ by the world's oceans provides a striking example of this, where increasing *p*CO_2_ can be both a resource *via* provision of limiting carbon (Connell, Kroeker, Fabricius, Kline, & Russell, 2013) to one organism, while simultaneously acting as a stressor to another organism *via* negative effects on carbonate chemistry and pH (Doney et al., 2012; Fabry, 2008; Ries, Cohen, & McCorkle, 2009). For basal producers such as algae and seagrasses, elevated CO_2_ concentrations have been shown to increase photosynthesis and growth when carbon sources are limiting (Harley et al., 2012; Koch, Bowes, Ross, & Zhang, 2013). Like other environmental drivers, however, this effect is nonlinear, and increasing plant performance begins to asymptote as other resources become limiting (Markelz, Strellner, & Leakey, 2011; Figure 2b, green line). In contrast, elevated CO_2_ and the resulting reduction in the pH of seawater (ocean acidification; OA) have negative implications for other organisms, particularly those that calcify (Ries et al., 2009; Kroeker et al. 2013). For many calcifying organisms, increasing *p*CO_2_ can display a threshold effect where small increases have a negligible effect, but the effects become increasingly severe past certain concentrations (Doney et al., 2012; Figure 2b, red line). Although not depicted in Figure 2, these responses can be highly variable among species (Fabry, 2008; Ries et al., 2009), and some shell‐forming organisms can display increased rates of calcification under elevated levels of *p*CO_2_ (Ries et al., 2009; Wood, Spicer, & Widdicombe, 2008). Noncalcifying organisms such as fish also exhibit threshold responses, but critical levels are generally much higher than for calcifiers (Ishimatsu, Hayashi, & Kikkawa, 2008; Munday, Crawley, & Nilsson, 2009).

Because no two species are likely to respond identically to any given environmental change in terms of performance, including their ability to defend themselves and to exploit or provide resources, environmental change will alter the outcomes of interspecific interactions. For example, factors such as body temperature can at some levels *increase* foraging rate (Sanford, 2002) but at higher temperatures can *decrease* foraging by the same species (Pincebourde, Sanford, & Helmuth, 2008). When changes in consumer feeding rates are not matched by changes in the production of resource species, indirect effects of environmental change can outweigh direct effects on lower trophic levels (Ghedini & Connell, 2017; O'Connor, Piehler, Leech, Anton, & Bruno, 2009). Environmental change can also disproportionately favor or disfavor species in competitive relationships. For example, primary producers that can rapidly respond to changing resources, for example nitrogen and carbon, will out‐compete habitat‐forming species which are slower to respond such as corals (Diaz‐Pulido, Gouezo, Tilbrook, Dove, & Anthony, 2011) and kelps (Falkenberg, Russell, & Connell, 2013; Gorman, Russell, & Connell, 2009). When foundation species or ecosystem engineers are sensitive to climate change (either positively or negatively), the distribution and abundance of other species may also change as a result (Crain, 2008; Crain & Bertness, 2006; Sunday et al., 2017). Finally, there can be important interspecific variation in the effects of climate change on phenology (Post, 2013), that is trophic mismatches (Edwards & Richardson, 2004; Post & Forchhammer, 2008).

## 4 THE PHYSIOLOGICAL BASIS OF ECOLOGICAL PHASE SHIFTS

**Figure 3:**
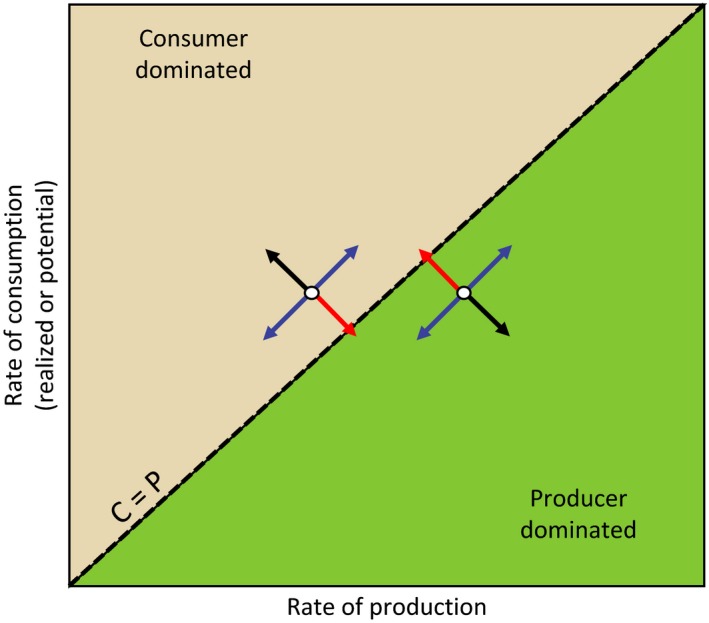
Conceptual diagram representing the balance between primary production and consumption in a two species system. The rate of production equals the rate of consumption along the dashed line. Where production exceeds consumption, producer biomass accumulates (green region). When consumption, or maximum potential consumption, exceeds maximum potential production, producer biomass is maintained at levels at or near zero (tan region)

Ultimately, the ecological impacts of climate change have physiological underpinnings that are subsequently mediated by interactions among species. One straightforward way to conceptualize the impacts of climate change on an interacting species pair is to first consider where their performance falls relative to one another under current environmental conditions, and then to examine how shifts in those conditions might affect the relative performance of the interacting pair (Figure 2). In so doing, we can identify suites of environmental conditions that may result in particularly rapid ecological change based on their relationship with inherent nonlinearities and potential tipping points in ecological systems (Connell et al., 2017; Kroeker et al., 2016; Monaco & Helmuth, 2011). To illustrate this, we consider a case within a simple food web consisting of one producer and one consumer (Figure 3); note that competition or other forms of interspecific interaction can easily be diagrammed in the same way if appropriate units are used to define the axes. In our system, there are two potential states: one where production outpaces consumption and “the world is green” (i.e., there is a high standing biomass of plants; Hairston, Smith, & Slobodkin, 1960), and one where instantaneous consumption rate, or maximum potential consumption rate based on standing consumer biomass, is higher than the rate of production and standing producer biomass is declining (instantaneously) or minimal (over the long‐run, barring ecological feedbacks to consumer populations) (Ling et al., 2015; Pace, Cole, Carpenter, & Kitchell, 1999). If our two species were a kelp and a sea urchin, one could envision these two states as a kelp forest and an urchin barren (Estes & Duggins, 1995).

This conceptual model allows us to explore how environmental change may alter the rate and timing of primary production, consumption, or both. Importantly, when environmental change confers equivalent benefits to both species, or equivalent costs to both species, the system tends to remain in the same state (blue arrows in Figure 3). When environmental change reinforces the status quo, the likelihood of a state change is reduced (black arrows). However, when environmental change disproportionately favors the species with the lower vital rate (either production or consumption), the balance between production and consumption can switch and the system can shift from one state to the other (red arrows). Note that the blue, black, and red arrows in Figure 3 correspond to regions in Figure 2 where environmental change causes the performance curves of the two species to move in parallel, diverge, or converge. Ecological examples of the potential phase shifts predicted by the red arrows include the widespread overconsumption of kelp forests that occurs in localities that accumulate high biomass of urchins, but kelp recovery occurs consistently when urchin biomass falls (Ling et al., 2015). Note that similar state shifts can occur when environmental drivers alter competitive scenarios; displacement of kelps by algal turfs occurs in localities that experience nutrient enrichment that disproportionately boost productivity and persistence of normally ephemeral turfs (Strain, Thomson, Micheli, Mancuso, & Airoldi, 2014). Superficially these ideas are similar to the mechanisms posited by the Metabolic Theory of Ecology (Brown et al., 2004), where consumer demand increases exponentially with temperature and mismatches among interacting species occur as the result of differences in scaling coefficients. An important distinction here is that because the relationship between photosynthesis or metabolism and temperature (i.e., a TPC) is not monotonic, it is possible for producers to be declining in productivity with increases in temperature, even while producers are increasing their demand, or vice versa. This is only possible if the biphasic nature of TPCs is considered, and cannot occur when metabolism and production are only considered to have an exponential relationship to temperature or other environmental driver.

## 5 SYNTHESIZING MULTIPLE, NONLINEAR DRIVERS IN MULTISPECIES SYSTEMS

**Figure 4:**
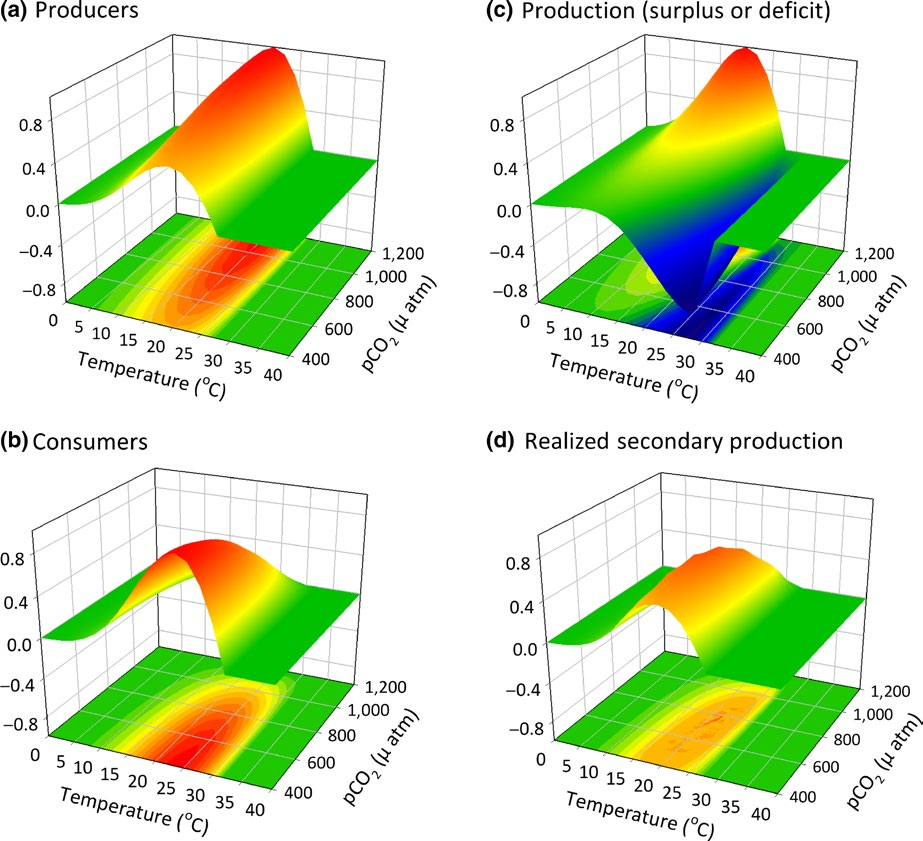
The cumulative effects of *p*CO_2_ and temperature on (a) the performance (productivity) of a hypothetical producer and (b) performance (grazing rate) of a hypothetical consumer where values (0–1) are scaled to the maximum. In this example, the optimal temperature of the producer is 24°C and that of the consumer is 20°C; the producer responds positively to increased *p*CO_2_ and the consumer responds negatively (see Figure 2). (c) In this scenario, at low levels of *p*CO_2_ and at temperatures close to the optimum of the consumer, the assemblage may experience a phase shift due to food limitation that occurs when the demand of the consumer outpaces productivity of the basal species. (d) The coupled direct (physiological) and indirect (food supply) effects on the consumer can also be calculated. Note that the ultimate consequences of temperature and *p*CO_2_ for consumers differ from what would be predicted by consumer physiology alone (compare (d) to (b))

One of the most difficult challenges in ecology is to understand ecological change as a function of its response to multiple, nonlinear factors via both direct (physiological) and indirect pathways (mediated by species interactions). Below, we present a framework to facilitate the exploration of these community‐level interactions based on response surfaces (Figure 4). We consider a hypothetical case with one primary producer and one consumer, as in Figure 3, where the two species exhibit different responses to two environmental drivers–temperature and ocean acidification–as diagrammed in Figure 2. Although we only explore two trophic levels, the approach can easily be extended to include multiple trophic levels (Provost et al., 2016). For simplicity, we consider the case where the interactions between the two stressors are multiplicative, which is likely an appropriate null expectation (Harvey, Gwynn‐Jones, & Moore, 2013; Sih, Englund, & Wooster, 1998). We can use these basic relationships to model a surface that represents the net rate of potential primary producer biomass change.

The combined effects of *p*CO_2_ and temperature will differentially alter producer and consumer physiological performance, as reflected by rates of production (Figure 4a) and consumption (Figure 4b). The difference between production and consumption determines net primary production rate when production is greater than consumption, and reveals production deficits where potential consumption is greater than the available production (Figure 4c). An additional response surface can be calculated to reflect the effects of temperature, *p*CO_2_, and food availability (producer or prey biomass) on the performance (biomass accumulation rate) of the consumer (Figure 4d). (We have assumed a starting consumer biomass of zero, but the model could easily be reconfigured to include negative values that represent consumer biomass loss when starting population size is positive but the energetic balance is unfavorable.) Comparing the surfaces in panels b and d helps to illustrate the conditions under which the constraints on consumer performance switch from resource limitation to physiological stress, assuming the simple scenario where stress effects are independent of resource availability (but see Schneider, Van Thiel, & Helmuth, 2010). Note that variable responses to OA and temperature (or to other combinations of abiotic drivers) among producers/prey and consumers may lead to ecosystem change *via* both direct and indirect effects. Consideration of the interspecific variation in physiological performance curves allows for a quantitative comparison of the assemblage‐level impacts of environmental change. For example, when the optimal temperature of a producer is higher than its consumer (Figure 4a,b), consumption can outstrip primary production at lower temperatures that are closer to the consumer's thermal optimum (trough in Figure 4c). At higher temperatures, closer to the optimal temperature of the producer, the opposite can occur and supply can exceed demand (Figure 4c).

**Figure 5:**
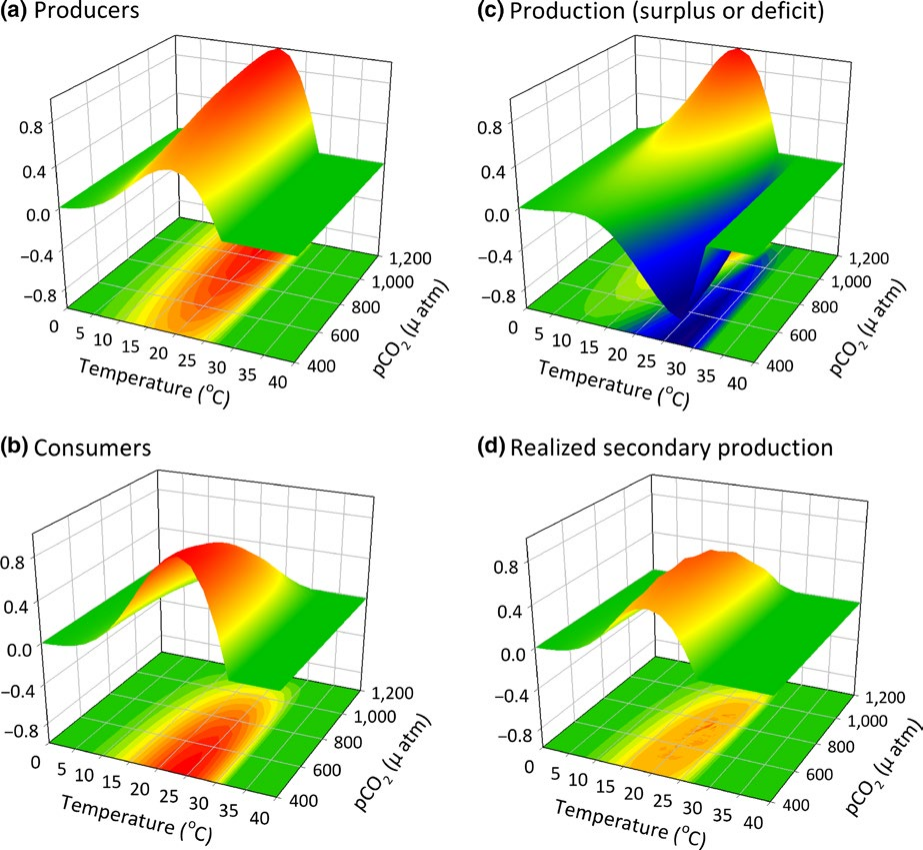
Performance of (a) producer and (b) consumer (c) resulting net primary production and (d) realized secondary production of the consumer in a scenario where the optimal temperature of the consumer (24°C) is higher than that of the producer (20°C). In this scenario, the system is fairly stable up to a temperature threshold above which sharp declines in net productivity occur

Surfaces such as these provide a means of quantitatively assessing the suite of conditions where direct physiological limitations on a consumer are likely to occur, and when effects are indirect *via* impacts on its resource. They also provide an initial estimate of the suites of environmental conditions under which ecological phase shifts are most likely to occur due to a change in net primary production. Regions of greatest instability, where any variability in drivers such as an increase in temperature may be most likely to cause a rapid shift in supply relative to demand, occur where the surface is steepest. In the example shown here (Figure 4c), under conditions where temperatures are close to the optimum of the consumer and levels of *p*CO_2_ are low, the system may reach a tipping point because there is insufficient production to meet the demands of the consumer (trough of negative production in Figure 4c). Nonetheless, when temperatures are slightly higher, closer to the optimum of the producer, and levels of *p*CO_2_ are high, the system exhibits a surplus of productivity because of depression of the consumer coupled with maximum production of the basal species. In scenarios where the producer has a lower thermal optimum than its consumer, the high producer biomass condition is instead stabilized at lower temperatures (Figure 5).

**Figure 6:**
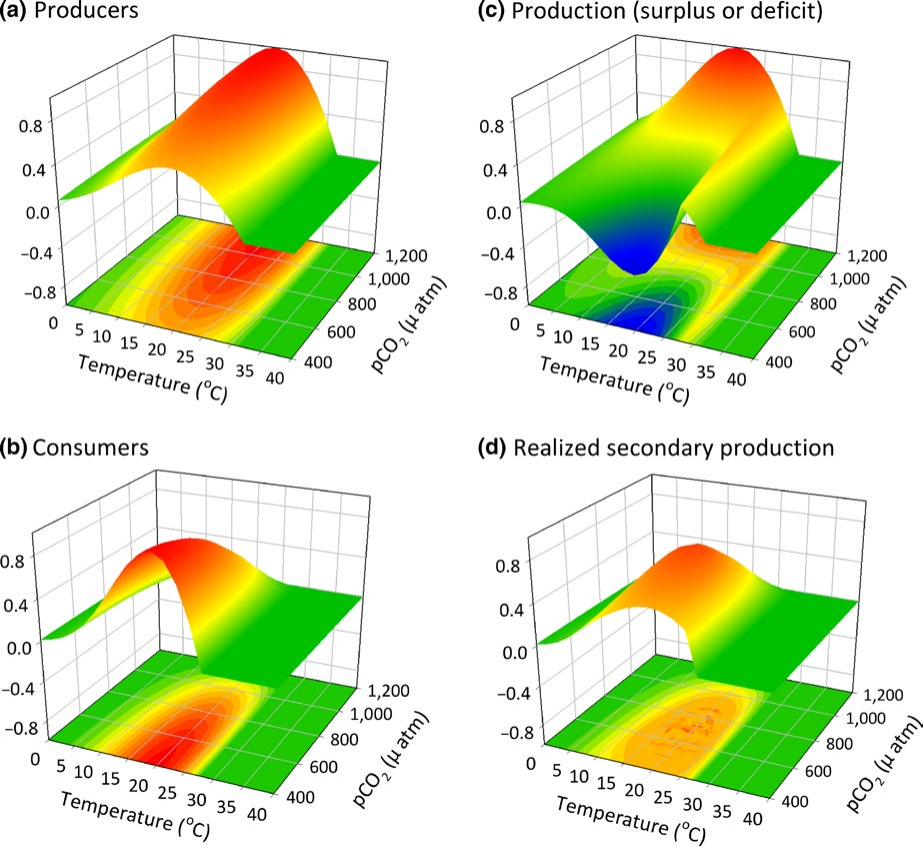
Performance of (a) producer and (b) consumer and resulting (c) net and (d) secondary production in a scenario where the optimal temperature of the consumer and producer are the same temperature (20°C) but the producer has a wider thermal performance breadth. In this scenario, the system is stable over a fairly wide range of conditions

**Figure 7:**
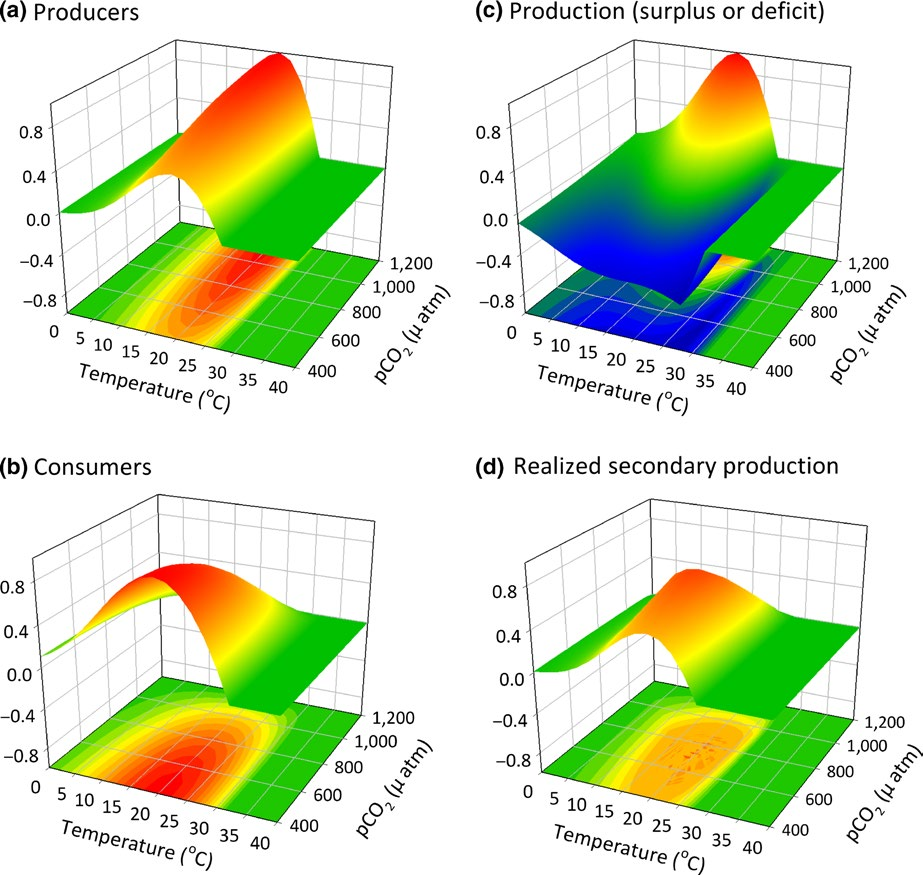
Performance of (a) producer and (b) consumer and resulting (c) net and (d) secondary production in a scenario where the optimal temperature of the consumer and producer are the same (20°C) but the consumer has a wider performance breadth. Under these conditions, the system is only stable under high levels of *p*CO_2_ where the producer does well and the consumer does not

**Figure 8:**
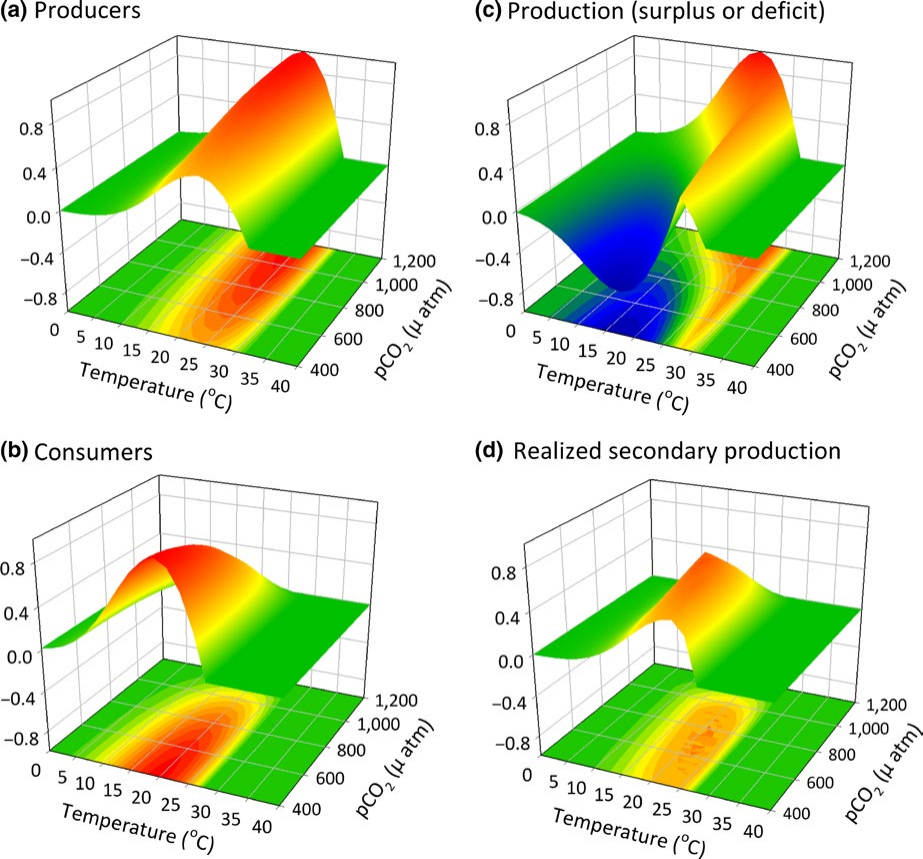
Performance of (a) producer and (b) consumer and resulting (c) net and (d) secondary production in a scenario where the optimal temperature of the consumer is lower than that of the producer (24°C) at low *p*CO_2_ but there is an interactive effect of *p*CO_2_ and temperature such that at high *p*CO_2_ the optimal temperature of the consumer shifts by 2°C lower, and the temperature at which foraging stops shifts by 4°C lower

Trophic mismatches in producer and consumer responses to environmental drivers often drive community shifts (red arrows in Figure 3), as seen with changes in phenology (Edwards & Richardson, 2004; Post & Forchhammer, 2008) or range expansions or increased abundance of warm‐adapted consumers (Ling, 2008). Yet, the physiological responses of organisms to environmental change can also stabilize community‐level properties (blue arrows in Figure 3) by driving individual responses (e.g., consumption) that aggregate to maintain stability (e.g., production). For example, enhanced primary production can allow herbivores to increase consumption rates and thereby maintain organismal processes (e.g., growth) across intensifying abiotic conditions (e.g., carbon and nitrogen release; Ghedini & Connell, 2016). Where resource supply mediates competitive dominance between key species (e.g., shifts from naturally kelp‐dominated to turf‐dominated systems), herbivores can counter these shifts by consuming the additional productivity of competing species (e.g., turfs; Ghedini, Russell, & Connell, 2015). This combination of direct (physiological and behavioral) and indirect factors (resource supply relative to demand) can contribute to the likelihood of resource limitation and hence stability of key components of communities (Ghedini & Connell, 2017) (Figures 6, 7, 8).

## 6 CONCLUSIONS

We considered the effects of two stressors by calculating the common currency of producer biomass (food energy; Sokolova, 2013) encompassing supply by the producer and demand by the consumer and demonstrate how variance among interacting species in their nonlinear responses to environmental change can be incorporated into predictions of community change or stasis. The scenarios presented are not meant to capture the full suite of conditions seen in nature. We focus on how trophic interactions (plant–herbivore) vary as a function of temperature and OA, and we do not delve into nonconsumptive effects (Matassa & Trussell, 2011; Matzelle et al., 2015) and or the potentially interactive effects of food supply and physiological tolerance (Matzelle et al., 2015; Schneider et al., 2010). Our approach seeks to move beyond more narrowly based definitions of drivers of change (i.e., stress and negative responses) to a more generalizable framework that recognizes the continuum of positive to negative changes in physiological performance and how their variance among strong interactors mediate community stability *via* both direct and indirect effects. Conversely, our framework also demonstrates the overarching importance of ecological context when interpreting studies on individual species. Feedbacks between these bottom‐up (direct effects of environmental change on producers) and top‐down processes (direct effects on consumers) are likely common, and argue for a further integration of studies at multiple levels of biological organization (Alcaraz, Felipe, Grote, Arashkevich, & Nikishina, 2014; Pawar et al., 2015).

If biologists are to inform climate adaptation strategies (Selkoe et al., 2015), then these physiological responses–both positive and negative–offer critical insights into the circumstances under which ecological phase shifts are most likely to occur (Harley & Paine, 2009; Wood et al., 2008). While several authors have noted the utility of quantifying differences in mortality (Case & Lawler, 2016), we know far less about how physiological processes and species interactions that occur under nonlethal conditions may result in large changes in ecosystem stability (Pfister et al., 2014).

In summary, we recognize the need for re‐aligning our conceptual frameworks that enable forecasts of ecological change. We reconcile positive with negative physiological responses to climatic and nonclimatic drivers and their underpinning of direct and indirect ecological responses. As research in ecological forecasting science intensifies, we call for embracing the nonlinear response of multiple species to multiple drivers and how variation among those *responses* elicits change in the interaction of species. By unifying organismal‐level responses with community‐level interactions we can thus move closer to anticipating and perhaps mitigating some of the inevitable effects of climate change.

## AUTHOR CONTRIBUTIONS

All authors contributed to the conceptual development, analytical simulations, and writing of the manuscript.

## CONFLICT OF INTEREST

None declared.
